# Cognitive Computing-Based CDSS in Medical Practice

**DOI:** 10.34133/2021/9819851

**Published:** 2021-07-22

**Authors:** Jun Chen, Chao Lu, Haifeng Huang, Dongwei Zhu, Qing Yang, Junwei Liu, Yan Huang, Aijun Deng, Xiaoxu Han

**Affiliations:** ^1^Baidu Inc., BeijingChina; ^2^The Affiliated Hospital of Weifang Medical University, Shandong, China; ^3^ National Clinical Research Center for Laboratory Medicine China; ^4^The First Affiliated Hospital, China Medical University, Liaoning, China

## Abstract

*Importance*. The last decade has witnessed the advances of cognitive computing technologies that learn at scale and reason with purpose in medicine studies. From the diagnosis of diseases till the generation of treatment plans, cognitive computing encompasses both data-driven and knowledge-driven machine intelligence to assist health care roles in clinical decision-making. This review provides a comprehensive perspective from both research and industrial efforts on cognitive computing-based CDSS over the last decade.*Highlights*. (1) A holistic review of both research papers and industrial practice about cognitive computing-based CDSS is conducted to identify the necessity and the characteristics as well as the general framework of constructing the system. (2) Several of the typical applications of cognitive computing-based CDSS as well as the existing systems in real medical practice are introduced in detail under the general framework. (3) The limitations of the current cognitive computing-based CDSS is discussed that sheds light on the future work in this direction.*Conclusion*. Different from medical content providers, cognitive computing-based CDSS provides probabilistic clinical decision support by automatically learning and inferencing from medical big data. The characteristics of managing multimodal data and computerizing medical knowledge distinguish cognitive computing-based CDSS from other categories. Given the current status of primary health care like high diagnostic error rate and shortage of medical resources, it is time to introduce cognitive computing-based CDSS to the medical community which is supposed to be more open-minded and embrace the convenience and low cost but high efficiency brought by cognitive computing-based CDSS.

## 1. Introduction

The Clinical Decision Support System (CDSS) has been widely accepted as a set of computer software programs that assists physicians and other health care roles in their decision-making whenever and wherever needed, like giving diagnostic suggestions based on the patient data or providing the knowledge about a specific disease. Compared to the type of CDSS that focuses on providing professional medical content, for example, UpToDate [1], there is a growing number of research and industrial efforts in cognitive computing-based CDSS [2]. Cognitive computing, as quoted in [3], “refers to systems that learn at scale, reason with purpose and interact with humans naturally.” Specifically, cognitive computing encompasses the advances of artificial intelligence including technologies like machine learning, natural language processing (NLP), computer vision, and speech recognition [3]. Despite that professional medical content providers like UpToDate, BMJ Best Practice, Cochrane Library, Embase, and DynaMed offer evidence-based content service for clinical decision support, it is inconvenient for health care roles to use actively and explicitly while taking care of patients. Besides, it is costly to manually summarize the diagnostic rules, manual decision trees, and treatment plans via plenty of clinical trials. In contrast with *deterministic* decision support based on human-readable medical knowledge, manually defined rules and decision trees, cognitive computing-based CDSS provides *probabilistic* clinical decision support via automatically learning and inferencing with large-quantity and high-quality clinical data. Due to the convenience, automation, and low cost of human labor, it is necessary to take advantage of cognitive computing-based CDSS to resolve the severe issues confronted by the current status of medical practice, including the following:

(i) The rate of misdiagnosis is high in primary health care facilities. According to an autopsy analysis over 50 years, the overall diagnostic error rate is 36.24% in China [4]. Due to severe lack of medical resources, the misdiagnosis rate in the primary health care facilities is estimated beyond the aforementioned number. Besides, there are approximately 12 million US adults who are misdiagnosed each year resulting in a misdiagnosis rate of 5.08% [5]
(ii) The lack of general practitioners is severe in primary health care. As the first protection of people’s health, the general practitioner is of great importance for primary health care. However, there exists a huge gap between the demands for general practitioners in society and the number of existing general practitioners. In China, a majority of medical students are trained to become medical specialists rather than general practitioners. The State Council of China has recently released a policy that by the year 2030, there should be at least 5 certificated general practitioners per 10,000 citizens, which means there is a lack of 500,000 general practitioners at least [6]. In the United Kingdom, the National Health Service has claimed a shortage of general practitioners [7] where the gap has been rising from 5,000 to 12,100 by 2020 [8]
(iii) The rapidly aging societies are faced with a lack of health care resources. Aging is one of the leading concerns in Asia where more than a quarter of the population will be over 60 years old by 2050 [9]. However, according to World Health Statistics released by the World Health Organization [10], the numbers of medical doctors per 10,000 population in China, Japan, Singapore, and Thailand are 19.8, 24.1, 22.9, and 8.1, respectively, compared to 36.8 in Australia and 35.9 in New Zealand among the western countries. There is an obvious and severe lack of health care resources in Asia, which can be fulfilled directly or partially fulfilled with the assistance of cognitive computing-based CDSS

Compared with CDSS focusing on providing human-readable medical knowledge or manually defined rules and decision trees, cognitive computing-based CDSS benefits from automatically learning and inferencing from a large amount of data with machine-readable medical knowledge, which is becoming the main stream of CDSS studies and industrial applications.

This review summarizes the major advances of cognitive computing-based CDSS in the last decade by discussing the characteristics, the general framework, and the applications as well as the limitations of cognitive computing-based CDSS.

## 2. Characteristics

Compared with other type of CDSS, cognitive computing-based CDSS is both *data*-driven and *knowledge*-driven [11]. Firstly, there are hundreds of information systems managing data of various modalities. It is natural that clinicians take multimodal data about a patient into account, e.g., texts of medical history from an Electronic Medical Record system and other medical contents from guidelines and eBooks, films of X-ray, CT and MRI scans from a Picture Archiving and Communications System (PACS), and tabular data from a Laboratory Information System. Cognitive computing-based CDSS automatically learns from large-scale multimodal data. Secondly, unlike general domains, medical knowledge is critical in health studies where the tolerance to decision errors is extremely low. Compared to human-readable medical knowledge in the medical content providers, cognitive computing-based CDSS requires the representation of *computable* medical knowledge. To transform human-readable knowledge to machine-readable knowledge is one of the core problems in cognitive computing-based CDSS, where various attempts have been made in the representation of medical knowledge.

### 2.1. Managing Multimodal Data

Cognitive computing-based CDSS relies heavily on clinical data where the volume, the quality, and the variety of data sources greatly affect the performance, the robustness, and the generality. Due to the sensitivity and privacy concerns, clinical data are rarely available before. With the desensitization technique, there are more and more publicly available multimodal clinical data like large-scale health data from intensive care admissions MIMIC-III [12] and eICU [13] as well as medical imaging data [14, 15] for researchers to experiment with. Cognitive computing-based CDSS deals with multimodal data, which can be categorized as

(i) *Clinical Text*. Most of the clinical notes are recorded with *texts*. To understand the clinical notes by machines, a preliminary task is the clinical Named-Entity Recognition (NER) [16– 18] which extracts medical entities of specific types like symptoms, vitals, and important findings in the reports and maps the entities to the predefined vocabulary. This enables machines to get the fundamental and critical keywords out of the pool of original texts. The pretrained language model [19– 22] is one of the main streams of the current NLP studies, which maps words (the minimum granularity of grammar) to universal high-dimensional vectors, namely, *embeddings*, by jointly training multiple NLP tasks on top of these embeddings. Based on medical entities and embeddings, many clinical problems are experimented with machine intelligence like giving diagnostic suggestions [23– 25], predicting clinical outcomes or codes [26, 27], and recommending medication combination [28].
(ii) *Medical Image*. Medical imaging is very common in clinical practice to assist the diagnosis and assess patient’s health conditions including X-ray film [29], CT film [30], MRI film [31], ultrasound image [32], fundus retina image [33], and pathological image [34]. Therefore, the machine is trained to automatically analyze the medical images to highlight the important findings in the images or give impression results. There are three main tasks that the machine usually tackles with computer vision models: detection [35– 37] (the existence of lesion and its location and size in the image), segmentation [31, 38] (outline the margins and edges of the lesions), and classification [30, 39] (figure out the class of the image or the lesion among the given classes). The technology has been applied in the analysis of medical images like lung cancer CT film [30], brain MRI film [31], fundus imaging for diabetic retinopathy [33], skin cancer imaging [39], and pathology [34].
(iii) *Tabular Data*. Tabular data are very common in the information systems from health care providers, e.g., demographic information of patients, laboratory test results, and order sets in the Computerized Physician Order Entry (COPE). Tabular data can be easily applied in machine learning models as a feature input. For example, demographic data and biomarkers from laboratory tests are used in the prediction of progression on patients with Alzheimer’s Disease [40]. It is straightforward to build decision trees or naive Bayes models on tabular data like the selection of the procedure in dental implant placement based on vertical ridge augmentation [41] as well as the prediction of heart disease [42].
(iv) *Audio Data*. Compared to text, image, and tabular data, audio data are less commonly used in the clinical workflow. A hearing test is a typical scenario that depends on the patient’s reaction to a pure-tone audiogram to determine the health condition of the patient [43]. By incorporating machine intelligence, it is able to automatically detect the dead regions of hearing on patients based on the hearing test [44]. With the technical advances of speech recognition, more attempts in cognitive computing-based CDSS have been made to automatically translate the clinical conversation between clinicians and patients from audio data to texts and further perform clinical decision support based on text analysis [45, 46].

In most cases, more than one type of data mortality can be analyzed together in real clinical procedures to comprehensively assess a patient’s health conditions. Therefore, cognitive computing is studied to manage multimodal data. For example, clinical texts are combined with tabular data in automatic diagnosis studies [23, 24, 47]. Medical images and clinical texts are fused for clinical diagnosis, prognosis, and treatment decisions [48]. Multimodal neuroimaging data are jointly analyzed in the study of Alzheimer’s Disease [49]. Audio data of the patient-doctor conversations are transformed into texts for better understanding about patient’s health [45, 46]. Besides, during the pandemic of COVID-19, the features of clinical texts, laboratory tests, and medical images on COVID-19 patients are systematically and jointly studied, which demonstrates a typical case of dealing with multimodal data. 

### 2.2. Computerizing Medical Knowledge

Medical knowledge, without a doubt, is critical in making clinical decisions. Compared with the human-readable knowledge, manually defined rules, and decision trees in the type of CDSS focusing on providing medical contents, cognitive computing-based CDSS studies how to make medical knowledge *computable* that is readable and manageable by machines. The studies of computable medical knowledge are generally divided into two groups: *explicit* and *implicit* knowledge representations. 

The studies of explicit medical knowledge representations are aimed at interpretable knowledge discovery on the large scale of medical data via learning patterns, associations, relations, pathways, and structures, which can be cognitively understood by machines. Below summarizes multiple types of approaches to explicitly computerize medical knowledge:

(i) *Regression Models*. Regression models are very common in clinical research. Given observations on the potential influential factors as well as their outcomes, regression models automatically fit to the observations via learning the weight of each factor which can further be used to predict the outcome of unseen samples. The weights obtained by regression models explicitly reflect how important each influential factor is in determining the clinical outcomes, which, apparently, is a perfect match with tabular data, for example, in the prediction of deaths due to COVID-19 [50]. Besides, multiple types of regression models are widely studied including a linear regression model for Alzheimer’s Disease diagnosis [51], a logistic regression model for cardiovascular risk prediction [52], a Poisson mixture regression model for heart disease prediction [53], and a polynomial regression model in the analysis of MRI films [54].
(ii) *Automatic Decision Trees*. Different from manually defined decision trees, automatic decision tree models like ID3 [55] and C4.5 [56] learn the logic of the decision process as in a rooted tree where each node corresponds to a specific observable variable, and the per-node branching strategy is generated by fitting to the observations with outcomes. The decision on a new sample is obtained by a walk from the root to one of the leaf nodes where the observation is matched with the branching conditions alongside the walk. Automatic decision trees have been widely studied in clinical decision support like in the prediction of obesity risk [57] and the study of differential diagnosis [58]. Based on the decision tree, ensemble learning is incorporated to improve the performance while preserving the feature of the decision tree. Examples include the gradient-boosting decision tree (GBDT) in the selection of important features to identify multicancer risk [59] and random forest in the prediction of cardiovascular risk [60] as well as an extreme gradient boosting in the prediction of smoking-induced noncommunicable disease [61] and the diagnosis of chronic kidney disease [62].
(iii) *Bayesian Methods*. The Bayesian methods are based on statistics and Bayes’ theorem, which explicitly represent medical knowledge using the estimated probability of a particular incident. In the simplest form, the naive Bayes model in the diagnostic suggestion problem [63] assumes that, if disease d can possibly cause symptoms $s_{i}$ or $s_{j}$ to be present on the patient with some probability, then the event that d causes $s_{i}$ to be present is independent of the event that d causes $s_{j}$ to be present on the same patient. Given that assumption, Bayesian methods estimate the probability of each disease based on the conditional probability that the disease causes each symptom to be present. In a real application, symptoms are not likely to be independent from each other, and the Bayesian network models are proposed to predict a diagnosis given a list of observations on symptoms, vitals, and other findings. Besides, Bayesian methods have been used in the prediction of osteonecrosis of the femoral head [64], the inference of diagnosis [65], the prediction of drug-induced liver injury [66], and the study of drug-target interaction [67] as well as those studies with Bayesian networks [23, 68].
(iv) *Medical Knowledge Graph*. Knowledge graph manages data in the graphical structure where the complex semantics are represented by concepts and relations. Apparently, medicine is a typical knowledge-driven subject compared with other domains like education or finance. Thus, the knowledge graph, which represents medical knowledge in an explicit and easy-to-access way, has been widely used in health care studies [69– 71], especially in cognitive computing-based CDSS. A knowledge graph mainly consists of ontology and relations. Ontology determines the form of conceptualization of the knowledge in a particular domain. Necessarily, an ontology contains the vocabulary of terms in the given domain and the specification of their meaning [72] as well as the interlinks between these concepts. Relations, often represented by triplets (*subject*, *predicate*, and *object*), elucidate how the entities in the knowledge graph are linked with each other. For example, the triplet *asthma*, *has_symptom*, and *coughing* means disease *asthma* has the symptom *coughing*. Once ontology is constructed, relations can be automatically extracted from various kinds of documents [73, 74]. For example, SNOMED CT is a typical resource of scientifically validated medical ontology where concepts, descriptions, and relationships are explicitly defined. With the complex semanticsrepresented by machine-readable concepts and relationships, the medical knowledge graph is widely used as external knowledge in predictive tasks for clinical decision support such as the classification of rare disease [75], the analytics of cancer data [76], and the prediction of patient’s health status [77].

The above lists the major approaches to explicitly represent medical knowledge in the ways that machine intelligence is able to process. In most cases, the medical knowledge graph can be combined with other approaches. For example, regression models and automatic decision trees depend on predefined features which are usually created by feature engineering, or alternatively, the definition of these features can be directly obtained from the medical knowledge graph.

Apart from the explicit representations, another group of methods to computerize medical knowledge is implicit representation or the more commonly used term *representation learning*. Compared with explicit knowledge, we classify the methods that focus on obtaining a computation-efficient and high dimensional latent feature vector for a given object, into the group of implicit representation. Generally speaking, implicit representation sacrifices interpretability in exchange for the performance gain in downstream tasks. Benefitting from the advances of deep learning, a great number of studies have been conducted in the representation learning of objects, widely known as *object*2vector, where *object* means the original form of human-readable knowledge. Examples of studies in medicine include *word*2vector [22, 78], *sentence*2vector [79], *document*2vector [80], *image*2vector, *speech*2vector [81], *EHR*2vector [82], and *patient*2vector [83]. 

There exist multiple ways to categorize the methods of representation learning. In this review, we split them into two groups based on whether or not external knowledge is considered in the representation learning of objects.

(i) *Direct Representation Learning*. This summarizes the group of methods that directly generates the representations of objects based on the original input without auxiliary external knowledge. A number of research works have been carried out on the representations of medical texts based on sequential models like the convolutional neural network [25, 27, 84] and the Gated Recurrent Unit [26] where clinical texts are processed as sequences of tokens and the sequential features are supposed to be captured in the representations. For the representations of medical images, studies have been performed on layer-wise representing images from local regions till aggregating to the representation of the whole image including both 2D images [85, 86] and 3D images [87]. Despite the above *supervised* methods that depend on the annotated labels of input to learn the representations, there exist *unsupervised* direct representation learning methods, generally known as an *autoencoder*, that encode the input into a feature vector in the latent space, and then, another network is used to decode the feature and reconstruct the input [88, 89]. By minimizing the difference between the original input and the reconstructed output, the autoencoder automatically learns the representations of the input without supervised labels.
(ii) *External Knowledge-Enhanced Representation Learning*. Studies have found that the performance of representation learning can be improved by incorporating external explicit knowledge, especially in the medical domain. There are various forms to introduce external knowledge in the representation learning including the description of medical concepts on Wikipedia Pages to enhance the implicit representation of diseases [90], the hierarchy from the International Classification of Diseases to update the representation of diseases [91] and the pathway to automatically infer the diagnosis [92], and the causal relationship between diseases and symptoms in automatic diagnosis [23, 24]. Besides, since multimodal data are widely used in cognitive computing-based CDSS, the incorporation of external knowledge as another kind of modality is very common. For example, by introducing explicit knowledge like risk factors extracted from patient questionnaires and an electronic health record, the performance of a deep convolutional neural network to represent breast cancer patients based on their mammography is significantly improved (AUC from 0.62 to 0.70) [93].

## 3. General Framework

**Figure 1:**
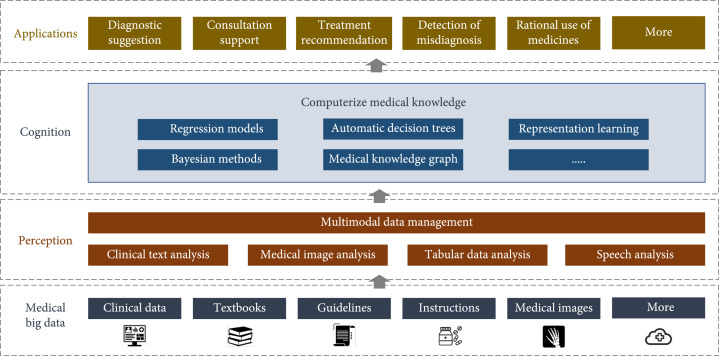
A general framework of cognitive computing-based CDSS. *Medical big data* manages the pool of heterogeneous medical data and provides the universal data access interface towards upper layers. *Perception* provides capabilities to process medical data of various modalities as well as holistic multimodal data analysis. *Cognition* computerizes medical knowledge with explicit and implicit methods, which enables machine intelligence to process medical data like humans. *Applications* provide various AI capabilities for clinical decision support based on the results from former layers. Examples include diagnostic suggestion, detection of misdiagnosis, and treatment recommendation.

According to the characteristics, we summarize a general framework to build a cognitive computing-based CDSS as shown in Figure 1. The framework consists of four layers: medical big data, perception, cognition, and applications.

(i) *Medical Big Data*. The effectiveness of most data-driven models is determined by the volume, the quality, and the variety of the data. Medical big data maintain a collection of large-scale high-quality medical data. For cognitive computing-based CDSS, there are usually three types of data: (1) Literature of human-readable medical knowledge including textbooks, guidelines, instructions, and research articles. (2) Clinical data that are generated in the information systems during the routine clinical events including clinical notes, medical films, laboratory test data, acoustic waveforms, and demographic data of patients. (3) Application data that are generated during the use of applications, e.g., system logs, clinicians’ feedback to the decision support, and statistical counts of different diagnosis codes. Medical big data meet the needs of data for training machine learning models, monitoring the status of CDSS and analyzing clinicians’ behaviors and preference as well as upgrading CDSS towards a better experience.
(ii) *Perception*. The perception layer provides functions to process the medical data of various modalities as well as the ability of a holistic multimodal data analysis. It integrates AI operators like natural language processing, medical image analysis, structured data analysis, and speech recognition to capture, understand, and manipulate various kinds of health-related data with respect to *language*, *vision*, *tabular data*, and *speech*. Moreover, the perception layer is able to jointly fuse the analysis on multimodal data.
(iii) *Cognition*. The cognition layer computerizes medical knowledge that transforms the human-readable knowledge from the perceived multimodal data into machine-readable knowledge with explicit methods like regression models, automatic decision trees, Bayesian methods, and medical knowledge graphs as well as implicit methods like representation learning. Computable medical knowledge which is learned from large-scale high-quality data forms the core of cognitive computing-based CDSS. It enables the downstream tasks to reason with medical knowledge. For example, the machine can read and understand the patient’s EMR documents under the assistance of clinical natural language processing that extracts symptoms, vitals, and other important findings and links them to the medical knowledge graph that shows what diseases these findings imply.
(iv) *Applications*. On top of the perception and the cognition layers, the layer of *applications* embeds the capabilities of CDSS into the clinical pathway of real practice. Some of the popular applications that cognitive computing-based CDSS has are diagnostic suggestion, treatment recommendation, consultation support, rational use of medicines, knowledge base for diseases, and treatment plans.

**Figure 2:**
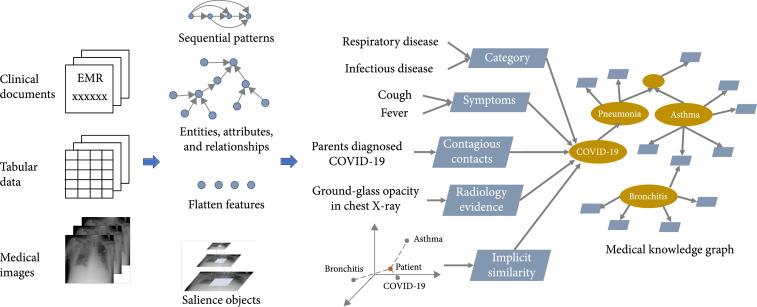
An example of diagnosis inference under the general framework of cognitive computing-based CDSS. Multimodal data about the patient are perceived by information systems. After extracting features and transforming to machine-readable medical knowledge, the features and knowledge acquired from different data sources are jointly analyzed to infer diagnosis.

Figure 2 demonstrates an example to automatically infer the diagnosis of the patient under the general framework of cognitive computing-based CDSS. Multimodal clinical data about the patient are simultaneously obtained including clinical documents like admission notes and medical records and tabular data like vitals and laboratory test results, as well as medical images like chest X-ray films. For clinical texts, sequential features are extracted by models like convolutional neural networks and recurrent neural networks, which can be used to classify the category of the diagnosis based on the text description, e.g., respiratory disease or infectious disease. Besides, critical entities, attributes, and relationships are extracted from clinical texts that add to the machine-readable knowledge about the patient, e.g., symptom *cough* is recognized in the texts. For tabular data, most of them can be directly used in machine learning models as flatten features except that some require extra processing. For example, continuous valued temperature from vitals may be discretized to *fever* or *not fever* and the count of white blood cell (WBC) per liter can be discretized to *low*, *normal*, and *high*. For medical images, deep learning models can be used to detect salience objects, segment lesions, and classify the types of lesions. All the above transform the human-readable medical data to evidences leading to the diagnosis of COVID-19, including the category, the symptoms, the history of contagious contacts, and the findings from radiology where the relationships and semantics are given by the medical knowledge graph. Besides, the similarity of the patient with COVID-19 can be measured by their implicit representations in the latent space. Therefore, multimodal data are jointly analyzed, and human-readable medical knowledge is transformed to machine-readable knowledge before generating the result of the diagnosis.

## 4. Applications

**Figure 3:**
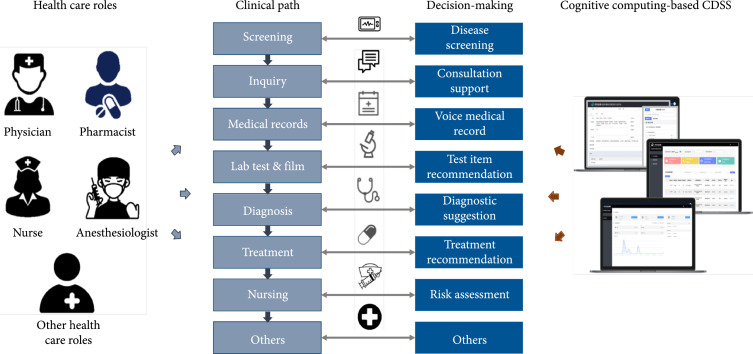
The overview of the general clinical pathway as well as the intervention points of cognitive computing-based CDSS. Throughout the clinical path, cognitive computing-based CDSS provides various functions for clinical decision-making. For example, consultation support guides physicians when inquiring with patients by suggesting questions to physicians. Voice medical record frees physicians’ hands by automatically recording and translating physicians’ voice data into the texts in EMR documents. Diagnostic suggestion shows the likelihood of the most probable diseases that the patient suffers according to clinical notes.

In this section, the applications of cognitive computing-based CDSS are reviewed. As illustrated in Figure 3, cognitive computing-based CDSS appropriately provides real-time decision supports to different health care roles like physician, pharmacist, and nurse at appropriate time through appropriate approaches alongside the routine clinical workflow. From the screening of disease throughout the clinical pathway to the discharge of patients, there are many points that cognitive computing-based CDSS is capable of supporting decision-making like consultation support, test item recommendation, diagnostic suggestion, and treatment recommendation. The following review some applications of cognitive computing-based CDSS along the clinical pathway.

### 4.1. Diagnostic Suggestion

Diagnostic suggestion is one of the commonly used applications. By jointly analyzing the clinical notes, reports, laboratory test results, and medical images, machine intelligence is capable of automatically inferencing the most appropriate diagnosis code, which is suggested to physicians before determining the ultimate diagnosis. In the study of pediatric disease diagnosis, the machine outperforms junior physicians (F1 score 0.885 vs. 0.840) in the diagnosis accuracy based on clinical notes, although being inferior to senior physicians (F1 score 0.885 vs. 0.915) [94]. By incorporating the knowledge of disease-symptom causal relationships in deep learning, Yuan et al. [24] achieves the state-of-the-art diagnosis results on the admission notes of the Top-50 most frequent diagnosis codes on the public MIMIC-III dataset [12]. In the study of skin cancer diagnosis based on visual skin images, the model based on convolutional neural network outperforms certificated dermatologists in the task of three-way classification, i.e., benign, malignant, or nonneoplastic, with average accuracy of 72.1% vs. 65.78% [39]. Moreover, in the study of the intraoperative diagnosis of a brain tumor based on stimulated Raman histology images, the performance of deep learning is superior to the pathologist-based interpretation with overall accuracy comparison of 94.6% vs. 93.9% [87]. These studies are evidences that in quite a few subjects, cognitive computing-based CDSS have been on par with human-level diagnostic performance. Diagnostic suggestions given by cognitive computing-based CDSS can actually assist clinicians in real-world diagnosis, especially in primary health care. Besides research results, diagnostic suggestion has been implemented as a critical feature of cognitive computing-based CDSS in industrial products including Baidu [23, 24] and iFLYTEK [95]. 

### 4.2. Detection of Misdiagnosis

Misdiagnosis is a severe worldwide issue in primary health care. Cognitive computing-based CDSS is capable of detecting diagnostic errors by comparing physicians’ diagnosis codes and features with those generated by a machine. A logistic regression-based study on the detection of delirium misdiagnosis for over 5 months on real medical records obtained an average accuracy of 72% [96], demonstrating an evidence that a machine can assist physicians in determining the error-prone diagnosis. Based on the medication data, there is a study that detects epilepsy patients out of medical records that are given nonepilepsy as diagnosis codes [97], which achieves AUC 97.2% on real medical records. 

### 4.3. Treatment Recommendation

There have been strong evidences that system-initiated recommendations of treatment to health care providers are effective at improving the quality of treatment orders [98]. Compared to diagnostic suggestions, treatment plans for frequent diseases are relatively fixed, which means treatment recommendation is more knowledge dependent. Therefore, the study of treatment recommendation in cognitive computing-based CDSS puts more focus on constructing the medical knowledge graph, e.g., the *disease-drug* bipartite graph [99], where treatment plans are induced once the diagnosis is determined. 

Besides knowledge-driven methods, there are many studies of data-driven models for treatment recommendation in cognitive computing-based CDSS. These models share similar assumptions that (1) horizontally speaking, the historical diagnosis and treatment data of all populations collectively demonstrate some patterns of cooccurrences, i.e., similar treatment for similar diagnosis. (2) Longitudinally, the patient’s past diagnosis and treatment data can influence the treatment on him/her in the near future. The study of recommendation of medication combination reduces the rate of Drug-Drug Interaction (DDI) by 3.6% from the existing EHR data [28], which lowers the risk for the patient to simultaneously take multiple drugs that should be avoided using together. There are many studies conducted on the public MIMIC-III dataset [12], which leverage the longitudinal medical records as well as the complex relationships between medications and diagnosis codes and achieve the performance of recommendation over 63% F1 score [100, 101]. 

### 4.4. Rational Use of Medicines

The World Health Organization, known as WHO, estimates that more than 50% of medicines worldwide are prescribed, dispensed, or sold inappropriately. The rational use of medicines requires the medicine to meet the patient’s need in an appropriate dosage and adequate period of time at a low cost with the minimum harm to the patient’s health. In a study on over 9 million prescriptions given by dentists [102], it is reported that about 96.6% of antibiotics are prescribed for irrational or uncertain indications, which leads to great waste of medical resources and unnecessary risk to patients’ health. 

With the ability to read and understand the instructions of medicines and the pharmacopeia as well as the patient’s clinical data, cognitive computing-based CDSS is capable of automatically detecting issues, e.g., error, warning, and conflict, in the prescriptions. These issues include potential DDI, medicine-allergy history conflict, medicine-demographic conflict (e.g., adult-only or pregnancy-only medicine), contraindication between diagnosis and medicine, medicine without corresponding compatible diagnosis, and medicine with inappropriate dosage, frequency, or period of time. The detection of potential DDIs is one of the hottest topics in this direction where the relationships of DDIs can be automatically extracted from texts with machine learning [103]. The review on the commercial DDI software reports the most commonly used one, Drug-Reax from Micromedex, due to its high sensitivity [104]. Drug Interaction Facts software for cancer patients is another widely used commercial DDI product that reports severity of DDI as well as the level of evidence to support the DDI with accuracy over 95% [104]. 

## 5. Examples of the Existing Systems

In China, cognitive computing-based CDSS has been implemented and deployed in many primary health care facilities to assist general practitioners in diagnosis and treatment. Among the providers of cognitive computing-based CDSS, Baidu, iFLYTEK, and Dr. Mayson are the representatives of the real systems in China. Each of these existing systems has advantageous features over the others. For example, based on the Chinese natural language processing and knowledge graph technology and products, the CDSS from Baidu is good at dealing with clinical texts [105] which is critical in cognitive computing like recommending diagnosis via understanding the patient’s illness from clinical texts [23, 24]. iFLYTEK is one of the top audio technology providers in China, and thus, the function of the *voice medical record* is a key feature in its CDSS, which frees the physician from manually typing and editing the texts in EMR. Instead, physicians interactively communicate with the computer via a customized microphone that captures the physician’s voice answer and translates to text data to automatically generate a medical record. Meanwhile, iFLYTEK has been the only system so far that passes the written test of the National Medical Licensing Examination in China in 2017 which surpasses 96.3% of human examinees [95]. It is a strong proof that the advance of cognitive computing-based CDSS has come to the state that has near-human performance and is ready to assist physicians in real medical practice. Besides, Dr. Mayson is empowered by the localization of the medical knowledge base from the Mayo Clinic that provides professionally verified descriptions about diseases and pathways as well as treatment plans.

**Figure 4:**
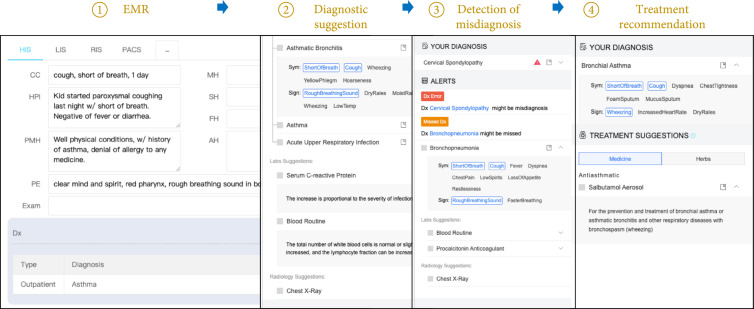
The demonstration of cognitive computing-based CDSS developed by Baidu. When a physician submits the clinical document in the EMR system, CDSS perceives the data and provides clinical decision support according to the stage that the physician is in. When illness description (e.g., chief complaint and history of present illness) is entered but the diagnosis is empty, CDSS provides diagnostic suggestions and highlights the symptoms or vitals matched from the suggested diagnosis to the contents of EMR. After physician enters a (list of) diagnosis, CDSS detects whether there is misdiagnosis or missed diagnosis and explains why it is very likely a misdiagnosis. The alert will disappear only when no misdiagnosis or missed diagnosis is detected at all. Then, the treatment recommendation will be given on the correct diagnosis.

Figure 4 demonstrates how cognitive computing-based CDSS is used in the medical practice, which is similar in the reviewed existing systems mentioned above. Firstly, cognitive computing-based CDSS works by integrating with other information systems like EMR, HIS, and LIS so that CDSS is capable of perceiving the clinical data. Multiple ways of integration are available including C/S (client/server), B/S (browser/server), and API. The choice of integration is influenced by the original information system that records the physician’s input because CDSS is not supposed to greatly change how the physician usually works or even induce an extra burden. To achieve the goal of appropriate information provided at the appropriate time in an appropriate way, cognitive computing-based CDSS will not present all relevant information at the same time. Instead, it only presents the supports to the current clinical decision with suggestions or system-initiated alerts. Therefore, cognitive computing-based CDSS has to recognize the scene and determines what supports that physician needs. An example is given in Figure 3.

## 6. Limitations

Despite the potentials to significantly decrease the errors, mistakes, and risks caused manually by health care roles in clinical activities, cognitive computing-based CDSS has obvious limitations which are great concerns before stepping into the spotlight of real medical practice.

(i) *The Habit of Cognitive Computing-Based CDSS*. There is a long way to go to help health care roles to develop the habit of using cognitive computing-based CDSS in the routine clinical workflow. The system is something new to clinicians who are still used to searching medical databases online or looking up in the books by hand in the old tradition when feeling uncertain in the diagnosis or treatment. It is necessary but challenging to widely introduce cognitive computing-based CDSS in real clinical activities and change the stereotype. Health care roles are supposed to be open-minded to embrace machine intelligence as their assistant.
(ii) *The Interconnection of Medical Data*. Due to the legal and privacy issues, the isolation of medical data between health care facilities has greatly limited the application of cognitive computing-based CDSS. The medical data are scattered in each single health care facility spreading across the nation. On the one hand, the isolation of medical data limits the performance of the data-driven methods in cognitive computing-based CDSS. On the other hand, it builds a barrier for machine intelligence to comprehensively understand the health conditions of patients. Therefore, it is important to push forward the interconnection of medical data from the administrative perspective.
(iii) *The Interpretability of Cognitive Computing-Based CDSS*. Despite the superior performance of machine intelligence compared with the traditional technology, the interpretability of results given by a machine has always been a controversial argument. In particular, most of the deep learning models are trained end-to-end as a black box. Different from other domains, the evidence for clinical decision support is necessary because it matters when dealing with every piece of health-related decision. There have been studies on the interpretability of machine learning models by incorporating attention mechanism [24, 26, 100] or external medical knowledge [23, 90]. More efforts are required in advancing the interpretability of cognitive computing-based CDSS

## 7. Conclusion

Over the last decade, cognitive computing has pushed forward the next generation of CDSS into real medical practice. Different from other types of CDSS, cognitive computing-based CDSS manages multimodal data and computerizes medical knowledge, which revolutionizes the way that machine intelligence assists health care roles in clinical decision-making. In spite of some reported use cases, the medical community is supposed to be more open-minded to work with cognitive computing-based CDSS as assistants, which is the new normal in the next decades.
